# A hybrid of regularization method and generalized path analysis: modeling single-vehicle run-off-road crashes in a cross-sectional study

**DOI:** 10.1186/s12874-023-02041-0

**Published:** 2023-10-06

**Authors:** Fatemeh Jahanjoo, Mohammad Asghari-Jafarabadi, Homayoun Sadeghi-Bazargani

**Affiliations:** 1 Road Traffic Injury Research Center, Tabriz University of Medical Sciences, Tabriz, 5167846311 East Azerbaijan Islamic Republic of Iran; 2 Cabrini Research, Cabrini Health, Malvern, VIC 3144 Australia; 3 Biostatistics Unit, School of Public Health and Preventative Medicine, Faculty of Medicine, Nursing and Health Sciences, Monash University, Melbourne, VIC 3004 Australia; 4 Department of Psychiatry, School of Clinical Sciences, Faculty of Medicine, Nursing and Health Sciences, Monash University, Clayton, VIC 3168 Australia

**Keywords:** Accident, Traffic accidents, Causal effect, Ridge regression, Lasso regression, Elastic net regression, Generalized path analysis

## Abstract

**Background:**

Determining risk factors of single-vehicle run-off-road (SV-ROR) crashes, as a significant number of all the single-vehicle crashes and all the fatalities, may provide infrastructure for quicker and more effective safety measures to explore the influencing and moderating variables in SV-ROR. Therefore, this paper emphasizes utilizing a hybrid of regularization method and generalized path analysis for studying SV-ROR crashes to identify variables influencing their happening and severity.

**Methods:**

This cross-sectional study investigated 724 highway SV-ROR crashes from 2015 to 2016. To drive the key variables influencing SV-ROR crashes Ridge, Least Absolute Shrinkage and Selection Operator (Lasso), and Elastic net regularization methods were implemented. The goodness of fit of utilized methods in a testing sample was assessed using the deviance and deviance ratio. A hybrid of Lasso regression (LR) and generalized path analysis (gPath) was used to detect the cause and mediators of SV-ROR crashes.

**Results:**

Findings indicated that the final modified model fitted the data accurately with ${\mathcal{X}}_{3}^{2}$= 16.09, *P* < .001, ${\mathcal{X}}^{2}$/ degrees of freedom = 5.36 > 5, CFI = .94 > .9, TLI = .71 < .9, RMSEA = 1.00 > .08 (90% CI = (.06 to .15)). Also, the presence of passenger (odds ratio (OR) = 2.31, 95% CI = (1.73 to 3.06)), collision type (OR = 1.21, 95% CI = (1.07 to 1.37)), driver misconduct (OR = 1.54, 95% CI = (1.32 to 1.79)) and vehicle age (OR = 2.08, 95% CI = (1.77 to 2.46)) were significant cause of fatality outcome. The proposed causal model identified collision type and driver misconduct as mediators.

**Conclusions:**

The proposed HLR-gPath model can be considered a useful theoretical structure to describe how the presence of passenger, collision type, driver misconduct, and vehicle age can both predict and mediate fatality among SV-ROR crashes. While notable progress has been made in implementing road safety measures, it is essential to emphasize that operative preventative measures still remain the most effective approach for reducing the burden of crashes, considering the critical components identified in this study.

## Introduction

Single-vehicle run-off-road (SV-ROR) crashes could include a vehicle that runs off the road and strikes a fixed object on the roadside (e.g., traffic sign, utility pole, tree, ditch, embankment, barrier, or culvert) or rolls over [1]. SVROR crashes account for a significant percentage of all fatal traffic accidents worldwide. For example, according to the statistical data released by the FHWA's Roadway Departure Safety Program, SV-ROR crashes accounted for 51 percent of all traffic fatalities from 2016 to 2018 in the United States [2]. In Sistan & Baluchestan, a province in South-East Iran, single-vehicle and rollover crashes accounted for 32.6% and 33.8% of all crash types between 2009 and 2010. There were also 4.80% fixed-object collisions [3]. ROR crashes are often more severe than other crash types When a vehicle crosses the center line and is involved in a head-on collision or leaves the roadway and collides with immovable roadside items. As stressed in the FHWA's Roadway Departure Safety Program, crossing a center line, Overturns, and hitting trees or shrubs on the roadside is the reason for more than 70% of ROR crashes [4]. In a study by Liu and Subramanian, ROR crashes accounted for 80.6% and 56.2% of crashes on highways and urban routes, respectively [5]. Consequently, it is important to develop an efficient method for investigating the unique characteristics and contributing factors of single-vehicle run-off-road (SV-ROR) crashes to reduce traffic injuries on highways. This would result from the fact that driving on highways is fundamentally different from driving in urban areas. Highways are more likely to have higher speeds and more instances of fatigue.

Several past studies have explored causal factors contributing to the occurrence and the consequent injury severity of ROR crashes. For example, Roque et al. evaluated the variables influencing the severity of ROR traffic crashes on freeways of Portuguese. Their empirical results highlight the problems with the present Portuguese road design, particularly regarding the requirements for providing forgiving slopes and justifications for installing safety barriers [6]. According to Liu and Subramanian, significant factors associated with a high risk of fatal single-vehicle ROR crashes include drunk-driving, curved road design, over-speeding, rural roads, high-speed limit roads, passenger cars, and unfavorable weather conditions [5].

In the analysis of crash severity, common statistical approaches such as regression models have been implemented in the long term because these models provide good indicators of the probability of an accident, and the results are interpretable. These standard approaches to statistical modeling require several assumptions about the underlying probability distribution of the data and pre-assumed relationships between the independent and dependent variables. If the assumptions are violated, biased estimates and incorrect inferences can be reached. Supplementary to this, although large population-based studies are frequently used to estimate predictors' effects in a real-world setting, they are susceptible to confounding bias for the lack of randomization. For this concern about randomization, methods from the causal inference framework have been explored as an approach for advancing robust and relevant science.

On the other hand, the number of variables to be entered in the causal modeling's conceptual diagram is always a problem, especially in traffic studies with many risk factors. First, it is difficult to identify true confounders concerning substantive knowledge alone. Also, ignoring a real confounder may lead to biased results, while including non-confounders can increase the variance. To address these challenges, Machine learning algorithms have been developed. Machine learning techniques have been proposed to solve problems in conventional statistical modeling. These techniques are now successful due to advances in computing power. These methods do not involve pre-defined relationships between study variables, and predictions are available without the need to understand the necessary mechanisms. Applied statistical methods and machine learning techniques overlap significantly because they deal with data analysis.

In this paper, we propose a hybrid method using machine learning techniques and path analysis to maintain sufficient number and efficient variables in the causal model of SV-ROR crashes. Beyond the methodological novelty, this study utilizes Haddon's matrix [7] to comprehensively analyze the phenomenon under investigation. Focusing variables on post-crash phases and human, vehicle, and environmental factors ensures a systematic and holistic approach to variable selection, capturing a wide range of factors that contribute to the occurrence and severity of the phenomenon. The study also focuses on establishing causal relationships to provide practical and applicable findings. By bridging the gap between interventional studies and real-world applicability, the research enhances understanding of how the studied variables influence the outcome, resulting in more accurate and meaningful findings. Additionally, the study addresses the traditional engineering perspective in traffic research and redirects the focus toward the health dimension of traffic. Moreover, considering the statistics of accidents and the severity of ROR crashes of a car, as well as the lack of sufficient information in this field in Iran (as far as we know), there is a need for clear and specific lines of accountability and improving the quality and quantity of technical resources available to all stakeholders to implement a safe system to reduce ROR crashes which can be addressed using the results of this study.

## Methods

### Study design and variables

This cross-sectional study includes information about 724 highway SV-ROR Crashes documented in Integrated Road Traffic Injury Registry System (IRTIRS) [8] from March 2015 to March 2016. All in all, there were 30 variables representing details of each crash in three main categories: i) crash scene-related, ii) Vehicle-related, and iii) driver-related information. The study's outcome, fatality, included two categories; non-fatal crash (Y = 0) and fatality as fatal crash (Y = 1).

### Ethics approval and consent to participate

This study used the information of people who entered the study voluntarily. Before participating in the study, all participants were given an informed consent form. Participants were assured that all their identifying information would remain confidential. This study was approved by the Research Committee under Protocol No. #1396.465 and the Ethics Committee under ethic No. #IR.TBZMED.REC.1398.1244of the Tabriz University of Medical Sciences.

### Statistical analysis

STATA (*Release 17*: *2021,* StataCorp LCC, College Station, Texas 77845–4512 USA) and M*Plus* (*Release 7.4: 2015*, Computer Software. Los Angeles, CA: Muthén & Muthén) software were used to conduct statistical analysis. In the initial step, the dataset maintained a ratio of 80% and 20% for training and testing sets, respectively. The proposed hybrid model used three machine learning regularization algorithms, namely: Ridge Regression (RR), Least Absolute Shrinkage and Selection Operator Regression (LR), and Elastic Net Regression (EnetR) for variable selection. The primary knowledge behind these models is the regularization of least squares through a regularization parameter *λ* [9].

For studying the regression model's regularization, it is necessary to solve optimization problems in norms terms. Accordingly, the ${\mathrm{L}}_{\mathrm{q}}$-norm for a real vector ***x*** ϵ ${\mathbb{R}}^{n}$ and q $\ge$ 1 is defined as the following:

$${||{\varvec{x}}||}_{q}{=\left(\sum\nolimits_{i=1}^{n}{\left|{x}_{i}\right|}^{q}\right)}^\frac{1}{q}$$

For q = 1 on obtains the L_1_-norm, and for q = 2 the L_2_-norm (called Euclidean norm as well). The L_2_-norm will be revisited while discussing RR and the L_1_-norm for LR.

### Ridge Regression (RR)

RR is a regularization method that benefits from regular least-squares approaches to minimize a loss fiction involving a sum of squared residuals. But in contrast to least squares methods, there is also a term called* λ* as *a* penalty parameter in the loss function, which is measured by the sum of squared regression weights. As indicated, this penalty parameter reduces the over-fitting and variability of the estimate by shrinking the weight of non-significant regression coefficients towards zero. RR minimizes the following function equation to estimate regression coefficients (*β*^) [10]:

$$\beta^{\wedge\mathrm{RR}}=arg\;\min\left\{\frac1{2\mathrm n}\sum\limits_{\mathrm i=1}^{\mathrm n}\left(y_{\mathrm i}\right.-\sum_{\mathbf j}\left.\beta_{\mathrm j}{\mathrm x}_{\mathrm{ij}}\right)^2+{\lambda\parallel\beta\parallel}_2^2\right\}$$

$$=arg\;\mathrm{min} \left\{\frac{1}{2\mathrm{n}}\mathrm{ RSS}(\upbeta)+\uplambda {||\upbeta||}_{2}^{2}\right\}$$

$$=arg\;\mathrm{min}\left\{\frac{1}{2\mathrm{n}} {|| \mathrm{y}-\mathrm{\beta X}|| }_{2}^{2}+\uplambda {|| \upbeta || }_{2}^{2}\right\}$$

Here the residual sum of squares (RSS) is called loss of the model, λ is the tuning, regularization, or penalty parameter which controls coefficients' shrinkage and λ$\parallel \beta {\parallel }_{2}^{2}$ is the tuning, regularization, or penalty term. The L_2_-norm $\left(\parallel\upbeta {\parallel }_{2}\right)$ is sometimes known as Tikhonov regularization.

### Lasso Regression (LR)

LR bears several parallels to RR since it also regularizes the loss function employing *λ* regularization parameter. Nevertheless, LR can choose the most significant independent variables and ignore those with negligible impact on the dependent variable. LR minimizes the following function equation to estimate regression coefficients (*β*^) [10]:

$${\upbeta }^{\wedge{\mathrm{LR}}}=arg\,\mathrm{ min}\left\{\frac{1}{2\mathrm{n}}\sum_{\mathrm{i}=1}^{\mathrm{n}}{({\mathrm{y}}_{\mathrm{i}}- \sum_{{\varvec{j}}}{\upbeta }_{\mathrm{j}}{\mathrm{x}}_{\mathrm{ij}})}^{2}+\uplambda {|| \upbeta||}_{1}\right\}$$

$$=arg\,\mathrm{min}\left\{\frac{1}{2\mathrm{n}} {|| \mathrm{y}-\mathrm{\beta X}|| }_{2}^{2}+\uplambda {|| \upbeta || }_{1}\right\}$$

Here λ is the tuning, regularization, or penalty parameter that must be estimated. It should be mentioned that there are some limitations to the LR estimator:

1. For *p* > n, the LR selects the most variables, which can be a limiting factor if the true model contains more than n variables.
2. For *n* > p and high correlation between predictors, the LR prediction performance is inferior to the RR.
3. The LR does not provide grouping property. In better words, it prefers to select just one variable from a group of highly correlated ones.

### Elastic net regression

EnetR is a combination of RR and LR. It is a highly effective algorithm since it uses two regularization parameters to combine the strengths and advantages of both RR and LR. EnetR minimizes the following function equation to estimate regression coefficients (*β*^) [10]:

$${\upbeta}^{\wedge{\mathrm{EnetR}}} =arg \mathrm{min }\left\{\frac{1}{2\mathrm{n}}{\sum }_{\mathrm{i}=1}^{\mathrm{n}}\left({y}_{\mathrm{i}}\right.-\sum_{\mathbf{j}}{\left.{\upbeta }_{\mathrm{j}}{\mathrm{x}}_{\mathrm{ij}}\right)}^{2}+ {\mathrm{\lambda P}}_{\propto }\left(\upbeta \right)\right\}$$

$$=arg \mathrm{min }\left\{\frac{1}{2\mathrm{n}} \parallel \mathrm{y}-\mathrm{\beta X}{\parallel }_{2}^{2} +{\mathrm{\lambda P}}_{\propto }\left(\upbeta \right)\right\}$$

$${\mathrm{Where P}}_{\propto }\left(\upbeta \right)= a\parallel\upbeta {\parallel }_{2}^{2}+\left(1 - \alpha \right)\parallel\upbeta {\parallel }_{1}$$

$$=\sum\nolimits_{\mathrm{j}=1}^{\mathrm{p}}{\mathrm{\alpha \beta }}_{\mathrm{j}}^{2}+ \left(1-a\right) \left|{\upbeta }_{\mathrm{j}}\right|$$

Here ${\mathrm{P}}_{\propto }(\upbeta )$ is the elastic net penalty term. For the particular case $\alpha$ = 1, it leads to the RR penalty, and for $\alpha$ = 0, the LR penalty. This penalty term is tuned out to be highly advantageous if *p* > n or when the predictors are highly correlated.

**Fig. 1:**
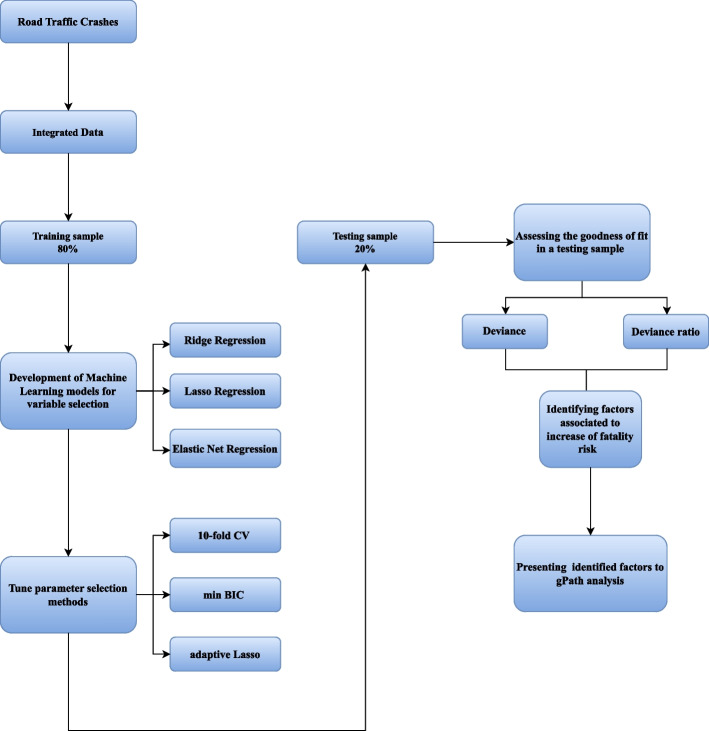
The overview of the proposed HLR–gPath model. Abbreviations Lasso: Least Absolute Shrinkage and Selection operator; CV: cross-validation; min BIC: minimum Bayesian information criteria; gPath analysis; generalized path analysis

The regularization models can deal with multicollinearity and be used for variable selection. The hyperparameters, namely *λ* and α in the models' elastic net, have been tuned through several procedures: tenfold cross-validation (CV), minimum Bayesian information criteria (min BIC), and adaptive lasso, wherever possible. Deviance and deviance ratios were used to assess the goodness of fit in a testing sample [10]. In the final step, to maximize the benefits of the algorithm in this hybrid approach, the output data from the advantageous method detected in the previous step with the selected variables were then presented to generalized path analysis (gPath). The gPath was performed to describe the direct and indirect relationships among a set of selected variables and enhance the prediction accuracy and interpretability of the results. There are six following steps in each path modeling [11]. Figure 1 illustrates an overview of the proposed HLR–gPath model.

1. Model specification

Model specification is necessary to detect relationships among a set of research variables. In this step, a graphical language, a practical and convenient approach to depicting complicated relationships, is used to design a conceptual model. The associations found in various studies are considered key clues and supporting information in constructing the conceptual model.

2. Model Identification

Model identification involves formulating the relationships mentioned in the model specification phase. Indeed it is related to the model to be fit to find the solution. It contains two conditions:

I. Rank condition: The rank condition is defined by the rank of the matrix, which should have a dimension (***M***-1), where M is the number of endogenous variables in the model. This matrix is formed from the coefficients of the variables (both endogenous and exogenous).
II. Order condition: This condition is defined by counting included and excluded variables in each equation. Although the rank condition tells us whether the equation under consideration is identified or not, the order condition tells us if it is precisely identified or over-identified.

3. Model estimation

The set of equations is simultaneously solved in the model estimation step to estimate the model fitting parameters. There are plenty of estimation methods namely: maximum likelihood parameter estimates with conventional standard errors and chi-square test statistic (ML), maximum likelihood parameter estimates with standard errors and a mean-adjusted chi-square test statistic (MLM), maximum likelihood parameter estimates with standard errors and a mean- and variance-adjusted chi-square test statistic (MLMV), maximum likelihood parameter estimates with standard errors and a chi-square test statistic (MLR), maximum likelihood parameter estimates with standard errors approximated by first-order derivatives and a conventional chi-square test statistic (MLF), Muthén's limited information parameter estimates with standard errors and chi-square test statistic (MUML), weighted least square parameter estimates with conventional standard errors and chi-square test statistic (WLS), weighted least square parameter estimates using a diagonal weight matrix with standard errors and mean-adjusted chi-square test statistic (WLSM), weighted least square parameter estimates using a diagonal weight matrix with standard errors and mean- and variance-adjusted chi-square test statistic (WLSMV), unweighted least squares parameter estimates (ULS), unweighted least squares parameter estimates with standard errors and a mean- and variance-adjusted chi-square test (ULSMV), generalized least square parameter estimates with conventional standard errors and chi-square test statistic (GLS), and Bayesian posterior parameter estimates with credibility intervals and posterior predictive checking (BAYES). Parameter estimates of the MLM and MLMV methods are robust to non-normality. The estimates of the MLR are robust not only to non-normality but also to non-independence of observations. The WLSM, WLSMV, and ULSMV methods use a full-weight matrix, and the GLS method uses a normal-theory-based weight matrix [12]. In this study, the WLSMV was used. This robust estimator does not assume a normal variable distribution and provides the best option for modeling categorical or ordered data [13].

4. Model testing

Path analysis supplies straightforward significance tests to determine the probable relationships between study variables, group differences, or the magnitude of explained variance. There is not a Straightforward test for deciding about model fit in path analysis. The best method for determining model fit is to examine several test outcomes. The collection of these numerous tests is known as model goodness of fit indices, which includes chi-square test/degree of freedom values ($\frac{{\mathcal{X}}^{2}}{\mathrm{df}}$) below five, root mean square error of approximation (RMSEA) values below 0.08, Tucker-Lewis index (TLI), and comparative fit index (CFI) values over 0.90. The chi-square test was used to conclude the significance of relationships, and *P*-values were calculated based on the statistics of this test.

5. Model modification

If the model's fit is unacceptable, it can be edited using significant modifications. Model modification entails changing an estimated and identified model by fixing free or freeing parameters that were fixed using modification indices provided by the model.

6. Model validation

The bootstrap and jackknife methods, which fall under non-parametric and resampling techniques, are used for model validation. Confidence intervals are generated using these two techniques for direct and indirect effects. In this study, bootstrap was used, and changes in percent between the width of the original model's confidence intervals and the ones resulted from the Bootstrap resampling method were calculated using the following formula:

$${P}_{change}= \left(\frac{{CI\;width}_{original\;model}- {CI\;width}_{Bootstrap\;method}}{{CI\;width}_{Bootstrap\;method}}\right) \times 100$$

We decided to set 15% as a predetermined amount so that models with a < 15% difference% according to this statistics would be considered model overlap, leading to suitable external validity.

## Results

### Main variables and measures

Among all the 724 SV-ROR Crashes, 101 (13.61%) were fatal. Overall, the information related to 28 explanatory variables was recorded. These explanatory variables are shown in Table 1 in more detail.

**Table 1:** Explanatory variables description in Single-vehicle Run-off-road Crashes (*n* = 724)

**Variable**	Variable level	Total crashes	Fatal crashes
		n (%)	n (%)
**Passenger presence**	no	321 (43.26%)	29 (28.71%)
	yes	421 (56.74%)	72 (71.29%)
**Crash day**	weekday	501 (67.52%)	65 (64.36%)
	weekend	241 (32.48%)	36 (35.64%)
**Lighting**	day	498 (67.12%)	72 (71.29%)
	night	218 (29.38%)	27 (26.73%)
	twilight/dawn	26 (3.5%)	2 (1.98%)
**Clear/cloudy weather**	no	41 (5.53%)	2 (1.98%)
	yes	701 (94.74%)	99 (98.02%)
**Dry road surface**	no	45 (6.06%)	2 (1.98%)
	yes	697 (93.94%)	99 (98.02%)
**Curved geometric design**	no	628 (84.64%)	82 (81.19%)
	yes	114 (15.36%)	19 (18.81%)
**Vehicle factor**	no	731 (98.52%)	99 (98.02%)
	yes	11 (1.48%)	2 (1.98%)
**Human factor**	no	196 (26.42%)	24 (23.76%)
	yes	546 (73.58%)	77 (76.24%)
**Collision type**	head-on collision	176 (23.72%)	38 (37.63%)
	rear-end collision	311 (41.91%)	41 (40.59%)
	T-bone collision	220 (29.65%)	11 (10.89%)
	side-swipe collision	35 (4.72%)	11 (10.89%)
**Road shoulder**	unpaved	35 (4.72%)	1 (0.99%)
	paved with soil	349 (47.04%)	47 (46.53%)
	paved with asphalt	358 (48.25%)	53 (52.48%)
**Road design**	one-way road	709 (95.55%)	100 (99.01%)
	two-way road	33 (4.45%)	1 (0.99%)
**Road defect**	no	702 (94.61%)	91 (90.1%)
	yes	40 (5.39%)	10 (9.9%)
**Permitted speed**	60–80	77 (10.38%)	1 (0.99%)
	80–95	38 (5.12%)	5 (4.95%)
	95–110	545 (73.45%)	83 (82.18%)
	110–120	82 (11.05%)	12 (11.88%)
**Vehicle type**	low	578 (77.9%)	83 (82.18%)
	high	153 (20.62%)	14 (13.86%)
	tricycle/ bicycle/motorcycle	11 (1.48%)	4 (3.96%)
**High-risk vehicle color**^a^	no	560 (75.47%)	72 (71.29%)
	yes	182 (24.53%)	29 (28.71%)
**Vehicle safety equipment**	low risk	456 (61.46%)	60 (59.41%)
	high risk	286 (38.54%)	41 (40.59%)
**Vehicle age**	less than 5yrs	261 (35.18%)	44 (43.56%)
	5 to 9 yrs	279 (37.6%)	34 (33.66%)
	10 to 14 yrs	154 (20.75%)	14 (13.86%)
	15 and more than 15yrs	48 (6.47%)	9 (8.91%)
**Vehicle plaque description**	personal regional	647 (87.2%)	90 (89.11%)
	other	95 (12.8%)	11 (10.89%)
**Vehicle maneuver**	forward	723 (97.44%)	100 (99.01%)
	turn	14 (1.89%)	0 (0%)
	other	4 (54%)	1 (0.99%)
	backward	1 (0.13%)	0 (0%)
**Driver fault status**	at fault	733 (98.79%)	98 (97.03%)
	not at fault	9 (1.21%)	3 (2.97%)
**Driver gender**	male	667 (89.89%)	94 (93.07%)
	female	75 (10.11%)	7 (6.93%)
**Driver education**^b^	illiterate	13 (1.75%)	3 (2.97%)
	primary	69 (9.3%)	7 (6.93%)
	nonacademic	595 (80.19%)	76 (75.25%)
	academic	65 (8.76%)	15 (14.85%)
**Driver job**	jobs with high economic status	642 (86.52%)	85 (84.16%)
	jobs with middle economic status	60 (8.09%)	14 (13.86%)
	jobs with low economic status	40 (5.39%)	2 (1.98%)
**Driver age (years)**^c^	Child (< 18)	1 (0.13%)	0 (0%)
	Adult (18 -65)	694 (93.53%)	92 (91.09%)
	Elderly (> = 65)	47 (6.33%)	9 (8.91%)
**Type of driving license**	class A	83 (11.19%)	7 (6.93%)
	class B	250 (33.69%)	33 (32.67%)
	class C	393 (52.96%)	58 (57.43%)
	motorcycle	6 (0.81%)	0 (0%)
	no license	10 (1.35%)	3 (2.97%)
**Driver seatbelt usage status**	used	525 (70.75%)	58 (57.43%)
	not used	217 (29.25%)	43 (42.57%)
**Driver Judiciary cause**	carelessness	725 (97.71%)	94 (93.07%)
	other	17 (2.29%)	7 (6.93%)
**Driver misconduct**	spiral movement	356 (47.98%)	6 (5.94%)
	over speeding	326 (43.94%)	66 (65.35%)
	other	60 (8.09%)	29 (28.71%)

^a^Low-risk colors: white, yellow, cream, pink, orange, brown; High-risk colors: silver, graphite gray, black, blue, green, dark blue, gray, purple, red [14]

^b^Primary: literacy and elementary education; non-academic: cycle, middle school, and diploma; academic: Bachelor's (B.Sc.), Associate's (A.Sc.), Master's (M.Sc.), and Doctorate (Ph.D.) degrees

^c^Age categories based on the driving regulations in the country

### Results of model selection methods for identifying risk factors of SV-ROR crashes

**Fig. 2:**
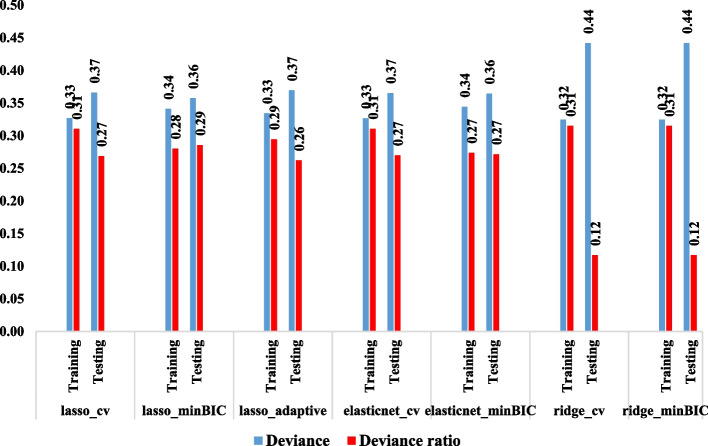
Results of model selection methods for identifying risk factors of SV-ROR crashes

As evident in Fig. 2, the LR and EnetR with min BIC method for selecting λ presented minor deviance. However, comparing deviance ratios revealed that LR outperforms EnetR with the largest Deviance-ratio. Based on the results from LR with min BIC λ selecting method, the presence of a passenger, collision type, vehicle age, and driver misconduct were identified as the main factors attributing to the severity of SV-ROR crashes. In the next step, these four variables were used to design the conceptual model and causal inference in the gPath model.

### Conceptual model

**Fig. 3:**
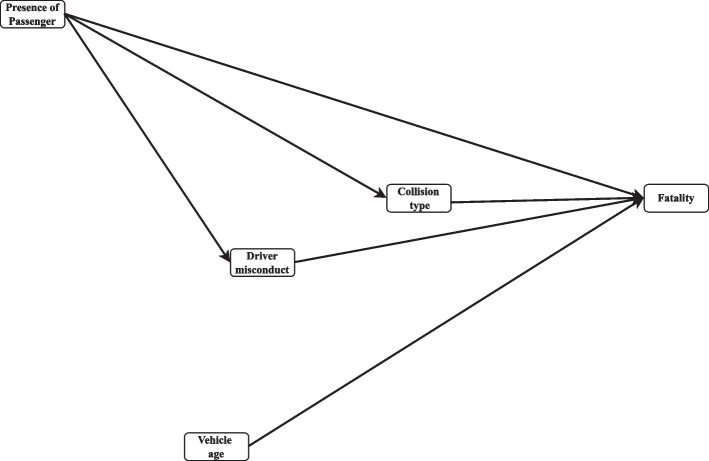
Hypothesized conceptual model

Figure 3 represents the hypothetical direct and indirect effects utilized for path analysis within the conceptual model. The relationships found in the numerous studies [15–21] were considered the key clues and supporting evidence to make this framework. In this model, fatality has been defined as the final endogenous variable (i.e., dependent). The presence of passenger and vehicle age have been defined as the exogenous variables (i.e., independent) and driver misconduct and collision type as the mediating variables. In Fig. 1, the arrows have been used to represent hypothetical direct effects, and the lines that comprise variables that simultaneously play exogenous and endogenous roles represent the mediating effects (e.g., collision type is an endogenous variable to passenger presence but an exogenous variable to fatality). With regard to present evidences the subsequent general objective is assumed:

A Hybrid of the Regularization method and generalized path analysis is a parsimonious model showing direct and indirect effects in modeling SV-ROR Crashes.

### Results of the hybrid LR-gPath model

**Fig. 4:**
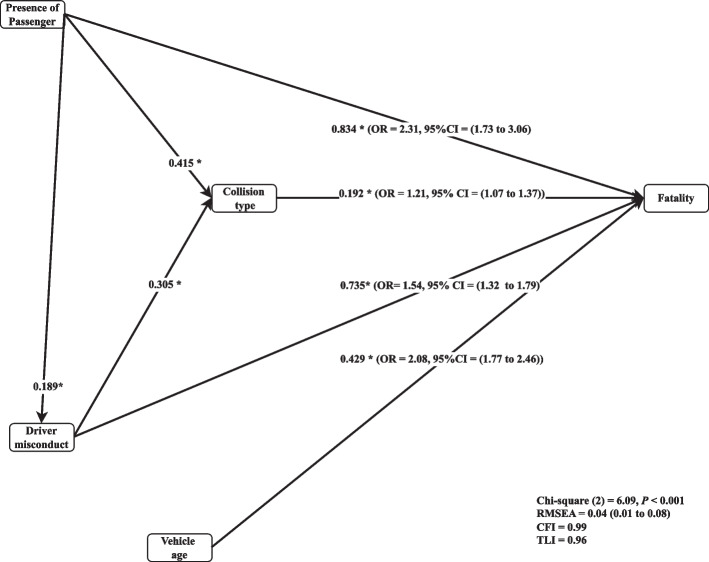
Perfect fitted model with standardized path coefficients *: *P* < 0.05

Five variables were in the hybrid LR-gPath (HLR-gPath) model; one variable was final endogenous, two were exogenous, and the other two were meditating. Initially, the model started with a conceptual model hypothesis that resulted in the following indices: with ${\mathcal{X}}_{3}^{2}$= 16.09, *P* < 0.001, ${\mathcal{X}}^{2}$/df = 5.36 > 5, CFI = 0.94 > 0.9, TLI = 0.71 < 0.9, RMSEA = 1.00 > 0.08 (90% CI = (0.06 to 0.15)). Based on modification indices and adding a path from driver misconduct towards collision type, the final full model fitted the data accurately with ${\mathcal{X}}_{2}^{2}$= 6.09, *P* < 0.001, ${\mathcal{X}}^{2}$/df = 3.05 < 5, CFI = 0.99 > 0.9, TLI = 0.96 > 0.9, RMSEA = 0.04 < 0.08 (90% CI = (0.01 to 0.08)). All conclusions hereinafter were drawn using the perfect fitted model (Fig. 4).

#### Direct effects

All coefficients on the perfect fitted model were statistically significant except at the 0.05 significance level. Findings showed significant direct relationship between fatality and following variables: passenger presence (odds ratio (OR) = 2.31, 95% CI = (1.73 to 3.06)), collision type (OR = 1.21, 95% CI = (1.07 to 1.37)), vehicle age (OR = 1.54, 95% CI = (1.32 to 1.79)), and driver misconduct (OR = 2.08, 95% CI = (1.77 to 2.46)).

#### Indirect effects

The mediated path model indicated that passenger presence (*β* = 0.003*;* 95% CI = (-0.007 to 0.013)) and driver misconduct (*β* = 0.108*;* 95% CI = (0.030 to 0.186)) had an indirect positive effect on fatality through their impacts on collision type. Furthermore, collision type (*β* = 0.181*;* 95% CI = (0.175 to 0.187)) was significantly and directly related to fatality through driver misconduct (Fig. 3).

#### Model validation utilizing the Bootstrap method

The changes in percent statistics between the width of the original model's confidence intervals and those resulting from the Bootstrap resampling method were in the range of 0.43 to 10.01. Considering the high overlap of the confidence intervals of the original model and the Bootstrap method, we assumed that the model has sufficient external validity.

## Discussion

This study is the first to discover that the cutting-edge HLR-gPath model's applicability may identify relationships and predict fatality in SV-ROR crashes. The proposed innovative HLR-gPath detects the most important risk factors in SV-ROR crashes and demonstrates the direct and indirect relationships between the selected variables. Traditionally, the gPath analysis has its own advantages when examining complicated relations among the many variables than having several independent variables with one dependent and comparing different assumed models against another to find which one best fits the used data. And LR can execute variable selection, leading to human effort and time-saving. In the proposed model in this study, the valuable variables were firstly extracted using LR, followed by the adaption of the gPath analysis to reveal stronger direct and indirect relationships to model SV-ROR crashes. Though our proposed hybrid model achieves promising results, it takes more processing time than standalone machine learning algorithms. In machine learning research, we aim to provide even better results with faster execution and greater effectiveness.

The findings from our study indicate a significant relationship between fatality and various factors, including the presence of passengers, collision type, driver misconduct, and vehicle age. We also observed that the presence of passengers influenced fatality through the mediation of driver misconduct and collision type. Furthermore, collision type was a mediator in assessing the relationship between fatality and driver misconduct.

In a similar local crash context, Yousefifard et al. (2021) [22] conducted a systematic review and meta-analysis to identify key risk factors contributing to road accident-related mortality in Iran. Their study included 20 studies involving 2,682,434 traffic accident victims and 23,272 deaths. The findings revealed that men had a 1.66 times higher risk of death than women, and each year increase in age raised the risk by 1%. Urban streets, roadway defects, not driving on flat and straight roads, and exceeding the speed limit were significant road-related mortality risk factors. Independent risk factors among road users included not maintaining focus on the road, not fastening seatbelts, and reckless overtaking. Pedestrians had a 2.07 times higher mortality risk than drivers and passengers. Accidents during daylight hours had a lower risk of death, while no significant relationship was found between mortality and vehicle types.

The authors of Yousefifard et al.'s study concluded that the limited number of studies on vehicle-related factors has resulted in a lack of comprehensive analysis of these important aspects. They also emphasized the absence of adjustment for key potential confounders in all the included studies, making it challenging to obtain a comprehensive and reliable understanding of the most crucial risk factors for road accident fatalities in Iran.

Our study focused specifically on single-vehicle runoff road crashes, which are known to be associated with higher risks and severity [23]. We observed a higher mortality rate than Yousefifard et al.'s study, which can be attributed to this specific focus. However, our study findings align with Yousefifard et al.'s study, demonstrating a consistent pattern of increased risk of death associated with several factors, including being male, advancing age, road defects, exceeding the speed limit, not maintaining focus on the road, failure to fasten seatbelts, and reckless overtaking.

It's important to note that our study did not include pedestrian data, a significant group in assessing overall mortality rates in road accidents. Therefore, caution should be exercised in interpreting the findings of our study. In contrast to Yousefifard et al.'s findings, our study revealed that accidents occurring during daylight hours were associated with a higher risk of death. This discrepancy in results may be attributed to factors such as increased traffic volume and higher speeds during the daytime, potential driver complacency or distraction due to better visibility, or variations in road user behaviors and characteristics specific to the studied population. Further research is necessary to explore and understand the underlying reasons behind this contrasting observation and its implications for road safety measures and interventions.

Our study addresses the knowledge gap highlighted by Yousefifard et al. in the context of local crash studies. We specifically focused on vehicle-related factors and aimed to account for crucial potential confounders lacking in previous studies. Our research contributes to a more comprehensive and reliable understanding of the most critical risk factors for road accident fatalities in Iran, considering the adjusted predictors. It's worth noting that Yousefifard et al.'s study, being a meta-analysis, covers the cumulative evidence up until 2021.

Here, we will expand the discussion on essential variables to provide a deeper and more comprehensive understanding of how they impact SV-ROR crash fatalities.

### The presence of a passenger impact on driver misconduct

The presence of passengers can exert both positive and negative effects on a driver's behavior and crash risk, contingent upon various factors [24]. Notably, previous studies have revealed that the presence of passengers can lead to reduced attention to driving and psychological pressure to drive less safely, particularly among young drivers [25–27]. Moreover, teenage passengers, particularly males, have been associated with an increased crash risk [27]. Passengers can distract drivers or encourage them to engage in risky behaviors, including speeding and other dangerous driving practices [28]. Similarly, it can be posited that having a passenger may induce elevated stress levels, thereby resulting in diminished driving proficiency [29].

Research has substantiated that conversing with passengers can distract drivers and serve as a predictor of driving misconduct [19]. A study conducted by Heck and Carlos sheds light on the various distractions drivers face when accompanied by passengers. Findings from this study indicated that, particularly for teenagers, engaging in conversation with friends in the vehicle can be distracting, and peers may even intentionally create hazardous situations due to the excitement or humor they derive from it. In addition to unwanted distractions from conversations or verbal exchanges, passengers can create further distractions for drivers by altering the radio, using drugs, physically interfering with or tickling the driver, or attempting to manipulate vehicle controls [30]. Moreover, another study identified the male gender, lower education levels, and an increased number of years of driving experience as factors predicting the occurrence of distinct distractions [31].

Nevertheless, it is crucial to acknowledge that the impact of passengers on driver behavior is not uniform across all drivers and passenger types [24]. In certain instances, passengers can offer a protective effect to drivers, thereby reducing their crash risk. For instance, drivers sometimes exhibit safer driving behavior when accompanied by passengers, and the presence of more passengers can diminish a driver's crash potential [32]. However, this protective effect is less pronounced among young drivers, during nighttime conditions, in situations involving slow traffic, and at crossroads [25].

Based on the outcomes of this study, it is plausible to consider the development of tailored driver education programs specifically addressing passenger distractions with the aim of reducing their frequency. In this context, cognitive psychology suggests that high-risk traveler behavior may represent a developmental norm that education can address.

### The presence of a passenger impact on collision type

The presence of passengers has a discernible impact on the type of collisions that may occur. Specifically, it has been observed that young drivers transporting only younger passengers are more prone to being involved in single-vehicle crashes transpiring under high-speed and low-volume conditions [32]. Such collisions, predominantly single-vehicle nature, render young drivers carrying passengers particularly vulnerable. However, for adult drivers, this collision type was found to be more injurious when the driver was unaccompanied in the vehicle [26]. A study conducted by Goel and Sachdeva sought to identify the causative factors, types, timing, and vehicle categories associated with crashes. Consequently, they ascertained that driver error predominantly contributes to head-on or rear-end collisions [33].

All in all, the presence of passengers has the potential to impact both driver misconduct and the specific type of collision that transpires. The influence exerted by passengers on driver behavior and crash risk is contingent upon various factors, including the age of the driver and passengers, as well as prevailing driving conditions. It is imperative for drivers to be conscious of these factors and take appropriate precautions to ensure the safety of themselves and their passengers on the road.

### Vehicle age

It is evident that older vehicles are generally less safe than their modern counterparts due to the lack of advanced safety features and improvements in crashworthiness. A study conducted by Ryb et al. [34] aimed to explore the relationship between the model year of vehicles and the risk of crash-related mortality for occupants. The analysis drew upon data from the National Automotive Sampling System Crashworthiness Data System (NASS-CDS) between 2000 and 2008. The study specifically focused on adult occupants seated in the front of vehicles. The findings unveiled that mortality rates decreased among later model year groups, indicating that newer vehicles were associated with lower crash-related mortality. After adjusting for potential confounding factors, it was observed that vehicles with model years ranging from 1994 to 2008 exhibited decreased odds of death compared to those with model years predating 1994. As a result, the researchers concluded that introducing newer vehicles into the automobile fleet likely contributed to the overall decline in mortality rates witnessed over the past two decades.

In a research article [35], the authors sought to investigate the impact of both aging drivers and vehicles on the severity of injuries sustained by vehicle occupants involved in road traffic crashes in Spain. The study findings revealed a linear escalation in crash severity with increasing vehicle age up to 18 years, after which the severity remained consistently at the highest level in bodily injury. Notably, no significant interaction was detected between driver and vehicle age regarding their impact on injury severity. These research outcomes hold particular significance for countries like Spain, where the driver population is experiencing extended longevity and the average age of vehicles on the road is progressively advancing.

Several reasons account for the heightened risk associated with older vehicles, including diminished crashworthiness (i.e., the ability of a vehicle to safeguard occupants during crashes), outdated safety devices that are more susceptible to failures due to aging, and the absence of contemporary safety features such as airbags, crumple zones, and electronic stability control. The correlation between newer vehicles and decreased crash-related fatalities is evidently established. Integrating these vehicles into the overall automotive fleet has played a substantial role in declining mortality rates. Consequently, the retirement of older cars from the fleet represents a pivotal step that undoubtedly contributes to a considerable reduction in crash-related deaths. This proactive measure underscores the importance of embracing advanced technology to enhance road safety.

### Strengths and weaknesses

The first limitation of the current study, there is not a precise and thorough registry system in the country to incorporate these data with hospital data and take them into account. The fact that accidents are likely not adequately reported to the authorities is another issue with this study. The study's strength can be seen in the fact that it took into account data from six of the country's most densely inhabited provinces, which allows for the generalizability of the findings. Another study's strength is introducing a hybrid model for modeling data from traffic crashes.

To further enhance the methodology for future research, it is crucial to acknowledge and address the limitations of this study. Firstly, we defined "traffic fatality" as deaths at the crash scene. However, it is essential to note that this definition was limited to deaths immediately at the crash scene. This limitation arose due to the lack of access to in-hospital data, which would have allowed us to include fatalities within 30 days of the time of the crash. Future research in this field must address this limitation and incorporate a more comprehensive assessment of traffic fatalities. Secondly, given the limited scope of this study, which only considered data from 2015 to 2016, it is crucial for future research to incorporate more up-to-date information. By extending the analysis period and including recent data, researchers will gain a deeper understanding of the evolving trends more accurately. This comprehensive approach will provide valuable insights into how we can further improve road safety measures moving forward.

This study is also limited to underreporting in road traffic crash studies, leading to incomplete and inaccurate data. It can result from official reporting mechanisms that fail to capture minor crashes or incidents without severe injuries or significant property damage. Additionally, individuals may choose to resolve minor crashes privately, bypassing official reporting channels. Social and cultural factors, including stigma and mistrust, further discourage reporting. To address underreporting, researchers and policymakers are suggested to utilize multiple data sources, such as police records, hospital records, and insurance claims, to ensure comprehensive data collection. Protecting confidentiality and anonymity encourages more accurate reporting, while public awareness campaigns can help overcome barriers and stigma associated with reporting incidents. By implementing these strategies and continuously monitoring and evaluating reporting systems, efforts can be made to minimize underreporting bias and improve the accuracy and comprehensiveness of road traffic crash studies.

## Conclusions

The introduced novel HLR-gPath model was practical in identifying rational crash pathways in SV-ROR crashes. They could predict fatality by Considering both exogenous and mediator variables simultaneously in a model. This study identified collision type and driver misconduct as mediator variables of SV-ROR crashes. It is suggested that when developing prevention strategies for SV-ROR crashes, health policymakers and healthcare professionals should consider the predominance of the mediators examined in this study.

## Data Availability

The dataset created and supported the current study's findings is not accessible by the general public since not requesting consent during the study protocol submission and from participants. However, they are available from the corresponding author upon reasonable request.

## Abbreviations

SV-ROR: Single Vehicle Run-Off-Road
IRTIRS: Integrated Road Traffic Injury Registry System
RR: Ridge Regression
LR: Least Absolute Shrinkage and Selection operator Regression
EnetR: Elastic Net Regression
gPath: Generalized Path Analysis
CV: Cross-Validation
min BIC: minimum Bayesian Information Criteria
$\frac{{\mathrm{x}}^{2}}{\mathrm{df}}$: Chi-square test/Degree of Freedom
RMSEA: Root Mean Square Error of Approximation
TLI: Tucker-Lewis Index
CFI: Comparative Fit Index
HLR-gPath: Hybrid Least Absolute Shrinkage and Selection Operator Regression Generalized Path Analysis
ML: Maximum likelihood parameter estimates with conventional standard errors and chi-square test statistic
MLM: Maximum likelihood parameter estimates with standard errors and a mean-adjusted chi-square test statistic
MLMV: Maximum likelihood parameter estimates with standard errors and a mean- and variance-adjusted chi-square test statistic
MLR: Maximum likelihood parameter estimates with standard errors and a chi-square test statistic
MLF: Maximum likelihood parameter estimates with standard errors approximated by first-order derivatives and a conventional chi-square test statistic
MUML: Muthén's limited information parameter estimates with standard errors and chi-square test statistic
WLS: Weighted least square parameter estimates with conventional standard errors and chi-square test statistic
WLSM: Weighted least square parameter estimates using a diagonal weight matrix with standard errors and mean-adjusted chi-square test statistic
WLSMV: Weighted least square parameter estimates using a diagonal weight matrix with standard errors and mean- and variance-adjusted chi-square test statistic
ULS: Unweighted least squares parameter estimates
ULSMV: Unweighted least squares parameter estimates with standard errors and a mean- and variance-adjusted chi-square test
GLS: Generalized least square parameter estimates with conventional standard errors and chi-square test statistic
BAYES: Bayesian posterior parameter estimates with credibility intervals and posterior predictive checking

## References

[1] AASHTO (2011). Roadside design guide.

[2] (FHWA) FHA. Roadway departure safety: Safety Federal Highway Administration. ; 2019. Available from: https://safety.fhwa.dot.gov/roadway_dept/.

[3] Rad M, Martiniuk AL, Ansari-Moghaddam A, Mohammadi M, Rashedi F, Ghasemi A (2016). The pattern of road traffic crashes in South East Iran. Global J Health Sci.

[4] (FHWA) FHA. Roadway departure safety: Safety Federal Highway Administration. ; 2014. Available from: https://safety.fhwa.dot.gov/roadway_dept/.

[5] Liu C, Subramanian R. Factors related to fatal single-vehicle run-off-road crashes. Department of transportation, national highway traffic safety administration. DOT HS 811 232. 2009. p. 1–25. Available from: https://trid.trb.org/view/913013/

[6] Roque C, Moura F, Lourenço CJ (2015). Detecting unforgiving roadside contributors through the severity analysis of ran-off-road crashes. Accid Anal Prev.

[7] Runyan C (1998). Using the Haddon matrix: introducing the third dimension. Inj Prev.

[8] Sadeghi-Bazargani H, Sadeghpour A, Lowery Wilson M, Ala A, Rahmani F (2020). Developing a national integrated road traffic injury registry system: a conceptual model for a multidisciplinary setting. J Multidiscip Healthc.

[9] Garefalakis T, Katrakazas C (2022). Data-driven estimation of a driving safety tolerance zone using imbalanced machine learning..

[10] Emmert-Streib F, Dehmer M (2019). High-dimensional LASSO-based computational regression models: regularization, shrinkage, and selection. Mach Learn Knowl.

[11] Tinsley H, Brown S (2000). Handbook of applied multivariate statistics and mathematical modeling:.

[12] Muthén LK, Muthén BO. Mplus statistical analysis with latent variables user’s guide. 7th ed. Los Angeles: National Institutes of Health; 2012.

[13] Brown T (2006). Confirmatory factor analysis for applied research.

[14] Jahanjoo F, Sadeghi-Bazargani H, Asghari-Jafarabadi M. Modeling road traffic fatalities in Iran’s six most populous provinces, 2015–2016. BMC Public Health. 2022;22:2234. 10.1186/s12889-022-14678-5 PMC9710022 36451170

[15] Duddu VR, Pulugurtha SS, Kukkapalli VM (2020). Variable categories influencing single-vehicle run-off-road crashes and their severity. Transportation Engineering.

[16] Gong L, Fan W (2017). Modeling single-vehicle run-off-road crash severity in rural areas: Accounting for unobserved heterogeneity and age difference. Accid Anal Prev.

[17] Austin R. Single-Vehcile Car Accidents 2022. Available from: https://romanaustin.com/clearwater-car-accident-lawyer/single-vehicles/.

[18] Sweetser DA (2007). Attitudinal and social factors associated with use of seat belts. J Health Soc Behav.

[19] Xiao Y (2020). Analysis of the influencing factors of the unsafe driving behaviors of online car-hailing drivers in china. PLoS ONE.

[20] Tseng C-M. Speeding violations related to a driver’s social-economic demographics and the most frequent driving purpose in Taiwan’s male population. Saf Sci. 2013;57:236–42.

[21] Simsekoglu Ö, Lajunen T (2009). Relationship of seat belt use to health and driver behaviors. Transp Res.

[22] Yousefifard M, Toloui A, Ahmadzadeh K, Gubari MIM, Madani Neishaboori A, Amraei F (2021). Risk Factors for Road Traffic Injury-Related Mortality in Iran; a Systematic Review and Meta-Analysis. Archives of academic emergency medicine.

[23] Okafor S, Adanu EK, Lidbe A, Jones S (2023). Severity analysis of single-vehicle left and right run-off-road crashes using a random parameter ordered logit model. Traffic Inj Prev.

[24] Regan MA, Mitsopoulos E (2001). Understanding passenger influences on driver behaviour: implications for road safety and recommendations for countermeasure development.

[25] Vollrath M, Meilinger T, Krüger H-P (2002). How the presence of passengers influences the risk of a collision with another vehicle. Accident Analys Prev.

[26] Orsi C, Marchetti P, Montomoli C, Morandi A (2013). Car crashes: The effect of passenger presence and other factors on driver outcome. Saf Sci.

[27] Simons-Morton BG, Ouimet MC, Zhang Z, Klauer SE, Lee SE, Wang J (2011). The effect of passengers and risk-taking friends on risky driving and crashes/near crashes among novice teenagers. J Adolesc Health.

[28] (NHTSA) National Highway Traffic Safety Administration. The effect of passengers on teen driver behavior. United State: 2012. Available from: https://www.nhtsa.gov/sites/nhtsa.gov/files/811613.pdf/.

[29] Meteier Q, Capallera M, De Salis E, Widmer M, Angelini L, Abou Khaled O, et al. Carrying a passenger and relaxation before driving: Classification of young drivers’ physiological activation. Physiol Rep. 2022;10(10):e15229. 10.14814/phy2.15229 PMC9115695 35583049

[30] Heck KE, Carlos RM (2008). Passenger distractions among adolescent drivers. J Safety Res.

[31] Lyu C, Ponce Jewell M, Cloud J, Smith LV, Kuo T (2019). Driving distractions among public health center clients: a look at local patterns during the infancy of distracted driving laws in California. Front Public Health.

[32] Lee C, Abdel-Aty M. Presence of passengers: does it increase or reduce driver’s crash potential? Accid Anal Prev. 2008;40(5):1703–12. 10.1016/j.aap.2008.06.006 18760099

[33] Goel G, Sachdeva S (2016). Analysis of road accidents on NH-1 between RD 98 km to 148 km. Perspect Sci.

[34] Ryb GE, Dischinger PC, McGwin G, Griffin RL (2011). Crash-related mortality and model year: are newer vehicles safer?. Ann Adv Auto Med Assoc Advance Auto Med Ann Scient Conf.

[35] Santolino M, Céspedes L, Ayuso M (2022). The Impact of Aging Drivers and Vehicles on the Injury Severity of Crash Victims..

